# Reprogramming of H3K27me3 Is Critical for Acquisition of Pluripotency from Cultured *Arabidopsis* Tissues

**DOI:** 10.1371/journal.pgen.1002911

**Published:** 2012-08-23

**Authors:** Chongsheng He, Xiaofan Chen, Hai Huang, Lin Xu

**Affiliations:** National Laboratory of Plant Molecular Genetics, Shanghai Institute of Plant Physiology and Ecology, Shanghai Institutes for Biological Sciences, Chinese Academy of Sciences, Shanghai, China; National Institute of Genetics, Japan

## Abstract

In plants, multiple detached tissues are capable of forming a pluripotent cell mass, termed callus, when cultured on media containing appropriate plant hormones. Recent studies demonstrated that callus resembles the root-tip meristem, even if it is derived from aerial organs. This finding improves our understanding of the regeneration process of plant cells; however, the molecular mechanism that guides cells of different tissue types to form a callus still remains elusive. Here, we show that genome-wide reprogramming of histone H3 lysine 27 trimethylation (H3K27me3) is a critical step in the leaf-to-callus transition. The Polycomb Repressive Complex 2 (PRC2) is known to function in establishing H3K27me3. By analyzing callus formation of mutants corresponding to different histone modification pathways, we found that leaf blades and/or cotyledons of the PRC2 mutants *curly leaf swinger* (*clf swn*) and *embryonic flower2* (*emf2*) were defective in callus formation. We identified the H3K27me3-covered loci in leaves and calli by a ChIP–chip assay, and we found that in the callus H3K27me3 levels decreased first at certain auxin-pathway genes. The levels were then increased at specific leaf genes but decreased at a number of root-regulatory genes. Changes in H3K27me3 levels were negatively correlated with expression levels of the corresponding genes. One possible role of PRC2-mediated H3K27me3 in the leaf-to-callus transition might relate to elimination of leaf features by silencing leaf-regulatory genes, as most leaf-preferentially expressed regulatory genes could not be silenced in the leaf explants of *clf swn*. In contrast to the leaf explants, the root explants of both *clf swn* and *emf2* formed calli normally, possibly because the root-to-callus transition bypasses the leaf gene silencing process. Furthermore, our data show that PRC2-mediated H3K27me3 and H3K27 demethylation act in parallel in the reprogramming of H3K27me3 during the leaf-to-callus transition, suggesting a general mechanism for cell fate transition in plants.

## Author Summary

Callus formation is a necessary step in regenerating a new plant from detached plant tissues, and the nature of the callus is similar to that of the root meristem. In this study, we intended to address the molecular basis that directs different plant tissues to form the root-meristem-like callus. We found that leaves and cotyledons, but not roots, of PRC2 mutants *curly leaf-50 swinger-1* and *embryonic flower2* lost the ability to form a callus. Using ChIP–chip analysis, we identified genes that were changed markedly in the histone H3 lysine 27 trimethylation (H3K27me3) levels during callus formation from leaf blades. Among these genes, a number of leaf-regulatory genes were repressed through PRC2-mediated H3K27me3. Conversely, certain auxin pathway genes and many root-regulatory genes were derepressed through H3K27 demethylation. Our data indicate that genome-wide H3K27me3 reprogramming, through the PRC2-mediated H3K27me3 and the H3K27 demethylation pathways, is critical in directing cell fate transition.

## Introduction

Unlike most animal tissues, a wide variety of plant tissues can easily acquire pluripotency when properly cultured *in vitro* with two plant hormones, auxin and cytokinin [1]. On a callus-inducing medium (CIM), explants usually first form a pluripotent cell mass, called callus, from which shoots and roots regenerate on the corresponding shoot- or root-inducing medium [2]. Recent studies revealed that callus formation is via a lateral root development pathway involving a process where cells evolve from pericycle-like to form root meristem-like cells [3]–[5]. This novel finding greatly improves our understanding of the plant cell regeneration process, but also raises a new question: what is the underlying mechanism that guides cell fate transition from diverse differentiated plant tissues to callus?

Callus formation from explants requires dramatic changes both in cell identities and cell growth patterns, and such a cell fate transition has been shown to be accompanied by changes in expression of numerous genes [3], [5]–[7]. It seems unlikely that the genome-wide changes in gene expression only involve spatially and temporally regulated transcription factors. Plant epigenetic pathways, which are known to influence genome-wide gene expression [8], may also participate in the large-scale gene regulations occurring during callus formation [7], [9].

Chromatin, which is composed of repeating units termed nucleosomes, is the template of epigenetic information, and changes in chromatin structure could lead to simultaneous expression changes of numerous genes [8], [10]. Changes of histone modifications often affect epigenetic regulation [11]. In *Arabidopsis thaliana*, silenced euchromatin is usually marked with histone H3 lysine 27 trimethylation (H3K27me3) [12]–[14], while the active gene transcription phase is usually associated with H3K4me3 and H3K36me3 [14]–[16]. We show in this work that reprogramming of H3K27me3 is critical in genome-wide regulation of key genes, leading to cell fate transition from leaf blade to callus.

Reprogramming of H3K27me3 involves the Polycomb group (PcG)-mediated H3K27 trimethylation and H3K27 demethylation. PcG proteins form at least two multiprotein complexes, namely Polycomb Repressive Complex 1 (PRC1) and PRC2. While PRC2 shows histone methyltransferase activity, targeting lysine 27 on the N-tail of histone H3, PRC1 recognizes the trimethylation marker for subsequent chromatin compression [17], [18]. The *Arabidopsis* proteins CURLY LEAF (CLF), SWINGER (SWN) and MEDEA [19]–[21] were proposed to be the core components of PRC2, and the H3K27 methyltransferase activity of CLF was shown in a biochemical assay [22]. In addition to the core components, PRC2 also contains other components, including EMBRYONIC FLOWER2 (EMF2) in *Arabidopsis* [21], [23]. On the other hand, RELATIVE OF EARLY FLOWERING 6 (REF6) is an H3K27me3 demethylase identified in *Arabidopsis*, which efficiently removes the methyl group from H3K27me3 both *in vitro* and *in planta* [24]. However, since the *ref6* mutation results in H3K27me3 hypermethylation only on a part of the PRC2-targeted loci [24], it is possible that other unidentified H3K27me3 demethylase(s) or additional demethylation mechanism(s) exist.

In this study, we show that genome-wide reprogramming of H3K27me3 is critically required for the leaf-to-callus transition. The PcG pathway is responsible for repression of the leaf-regulatory genes in leaf blade explants, and acts in parallel with the *Arabidopsis* H3K27 demethylation pathway, which derepresses the auxin-pathway and root-regulatory genes to enable the leaf-to-callus transition.

## Results

### Mutations in PRC2 genes block callus formation from leaf blades

**Figure 1:**
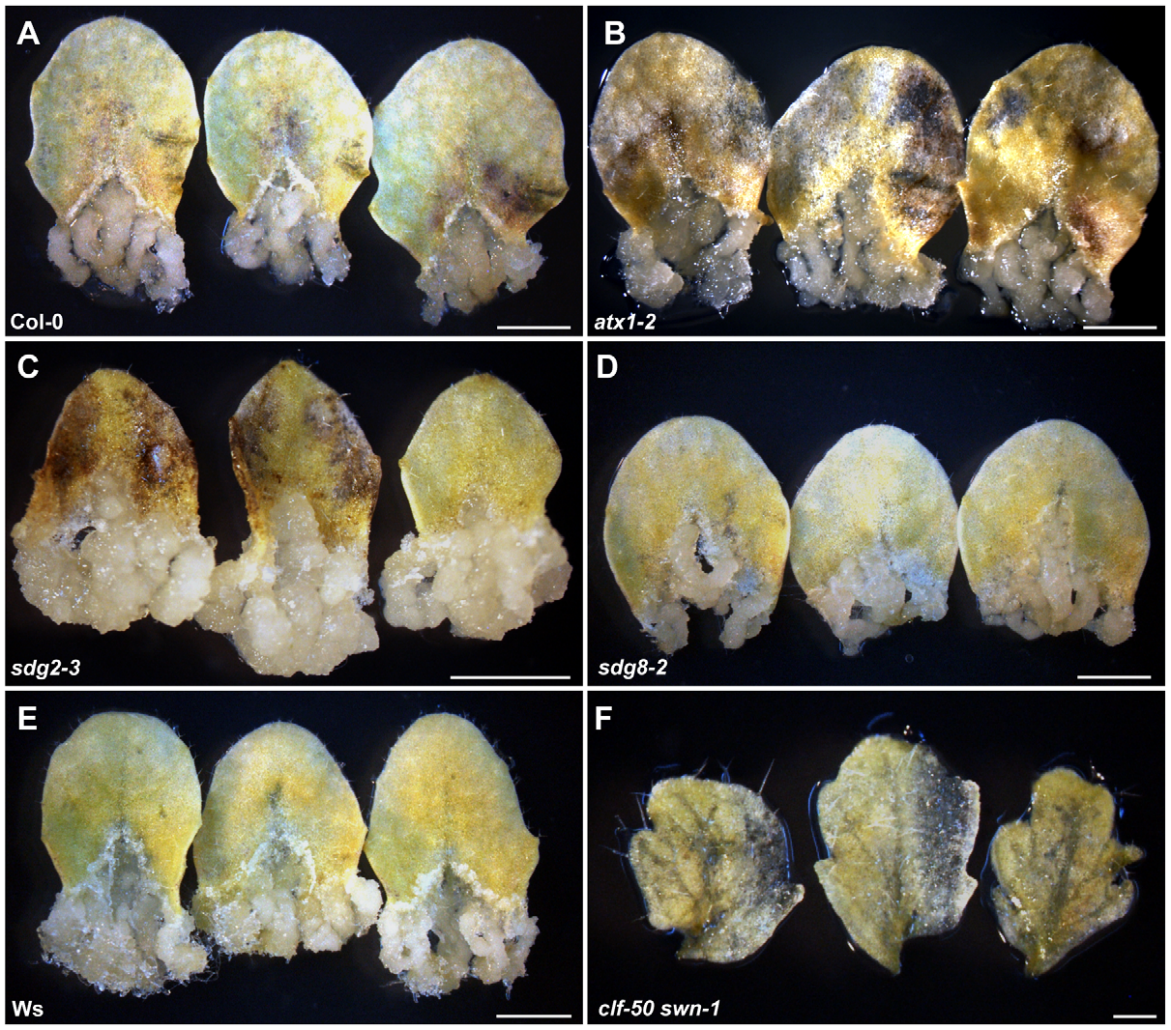
Leaf explants of the PcG double mutant *clf-50 swn-1* lose the ability to form a callus. Sterilized seeds were grown on plain MS media. The third and fourth rosette leaves of 20-day-old seedlings were cut at the junction between the blade and the petiole and only the blade parts were cultured on CIM for another 20 days. (A–F) Wild-type Col-0 (A), *atx1-2* (B), *sdg2-3* (C), *sdg8-2* (D), wild-type Ws (E), and *clf-50 swn-1* (F). Note that only leaf explants, which were from the *clf-50 swn-1* rosette leaves, failed to form callus. For each genotype, more than 30 rosette leaves were used in one experiment, and they all exhibited the consistent phenotype. Shown are three randomly picked leaves out of the 30 tested leaves for each genotype. Bars = 2 mm in (A–E) and 400 µm in (F).

Because the gene expression profiles in calli differ dramatically from those in their original tissues [3], [5]–[7], we hypothesized that one or more epigenetic pathways, which function in genome-wide regulation of gene expression, may participate in this cell fate switching process. To test this hypothesis, we first analyzed callus formation using leaf blades from mutants with reduced levels of H3K4me3, H3K36me3, or H3K27me3, which in *Arabidopsis* are important for gene regulation in the euchromatin regions [11], [14]. For convenience, explants of leaf blade are referred to as leaf explants hereafter. The mutants used corresponded to the methyltransferases of ARABIDOPSIS THALIANA TRITHORAX1 (ATX1) and SET DOMAIN GROUP2 (SDG2) for H3K4me3 [25]–[27], SDG8 for H3K36me3 [28], [29], and CLF and SWN for H3K27me3 [12]. Compared with the wild type (Figure 1A, 1E), *atx1-2*, *sdg2-3*, and *sdg8-2* leaf explants formed calli on CIM (Figure 1B–1D), whereas no callus was seen from leaf explants of the *clf-50 swn-1* double mutant (Figure 1F). It should be mentioned that *swn-1* is a weak allele, and the null *clf swn* double mutant displays distinct plant phenotypes [21]. Because both CLF and SWN are core components of PRC2 and are functionally redundant, these results suggest that the PRC2-mediated H3K27me3 is required for the leaf-to-callus transition.

### Genome-wide changes in H3K27me3 distribution in the leaf-to-callus transition

**Figure 2:**
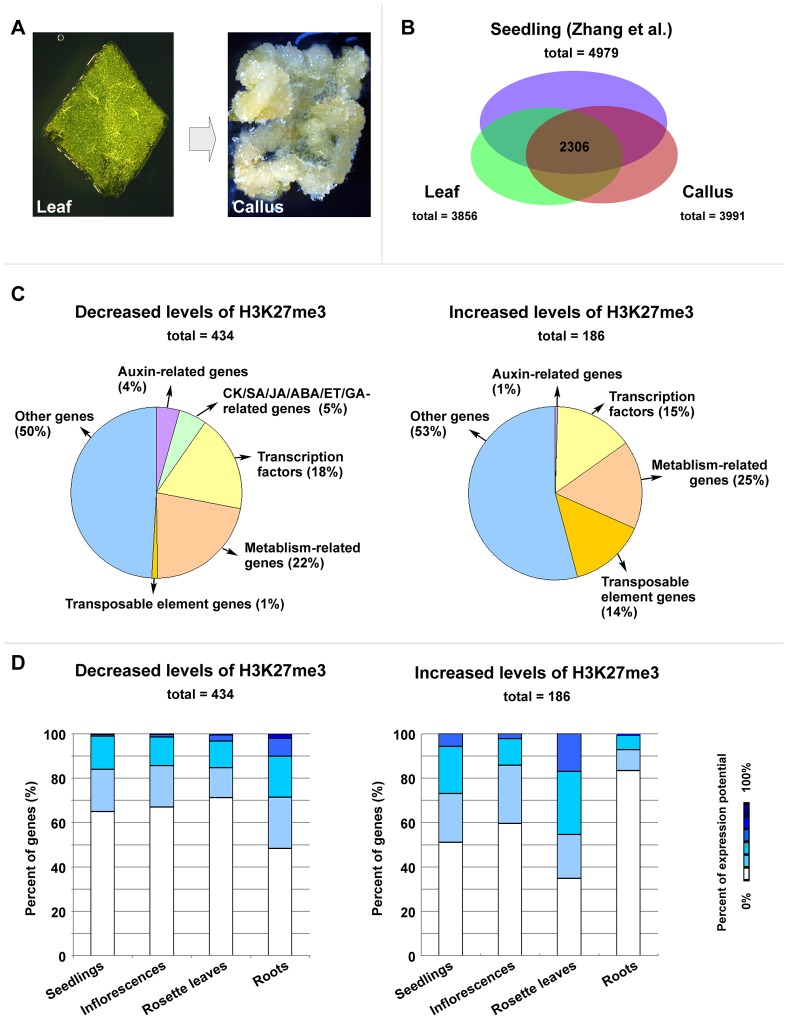
ChIP–chip analysis to identify genes with changes in the H3K27me3 levels between leaf and callus. (A) Leaf explants (left) and calli (right) were used for the ChIP-chip experiment. For preparation of experimental materials, leaf explants that were just cut from leaves of 20-day-old wild-type Col-0 seedlings and calli that were on the margins of leaf explants cultured on CIM for 20 days were harvested for nuclei extraction. (B) A comparison of H3K27me3 covered loci taken from three independent genome-wide analyses (seedlings from Zhang et al. [13], and leaves and calli in this work). (C) Classifications by annotated functions of the identified H3K27me3-covered genes with either decreased (left) or increased (right) levels. (D) Expression profiles of the genes identified by the ChIP-chip experiment with the decreased (left) or increased (right) H3K27me3 levels. The original expression data were downloaded from the online program Genevestigator [30].

Since H3K27me3 is required for callus formation from leaves, we next analyzed the genome-wide H3K27me3 profile by a ChIP-chip assay to identify genes with the altered H3K27me3 modification between the leaf and callus (Figure 2A; Table S1). A total of 3856 and 3991 genes harboring high levels of H3K27me3 were identified in the leaf and callus, respectively, with many of these included among the 4979 genes previously identified in seedlings [13] (72.7% and 75.6%, respectively) (Figure 2B). Additionally, 2306 genes were common among the leaves, calli, and seedlings (Figure 2B). Furthermore, 434 and 186 genes showed considerable and significant decrease and increase in H3K27me3 levels, respectively, in the callus as compared to the leaf (Figure 2C, Table S1).

Genes bearing the altered H3K27me3 levels encode proteins with different functions (Figure 2C), including phytohormone pathway proteins and putative transcription factors. It is possible that some of these putative regulatory genes may have important roles during callus formation, and we thus analyzed expression profiles of these putative regulatory genes in four different tissues: seedlings, inflorescences, rosette leaves, and roots, based on the data from published databases [30]. Compared with leaves, calli contained the increased number of genes which are normally expressed in the root of a plant, and all these genes belonged to the category with decreased H3K27me3 levels (Figure 2D, left). Conversely, among the H3K27me3 level increased category, the number of genes normally expressed in the root of a plant was reduced, whereas the number of genes expressed in the leaf was increased (Figure 2D, right). Since the H3K27me3 modification is generally thought to negatively affect gene expression [12], [13], these results support the previous notion that the callus cells are the root-featured cells [5].

We also performed a microarray experiment to analyze whether and to what degree the changed H3K27me3 levels of these genes affect their expression levels. Our data showed that about 40% genes with decreased H3K27me3 levels were upregulated, and about 46% genes with increased levels of H3K27me3 were downregulated (Table S2). This result indicates that the H3K27me3 modification and gene expression regulation are correlated for a relatively large group of the H3K27me3-covered genes during the leaf-to-callus transition.

### Phenotypic analyses of leaf explants reveal two distinct stages of callus formation

**Figure 3:**
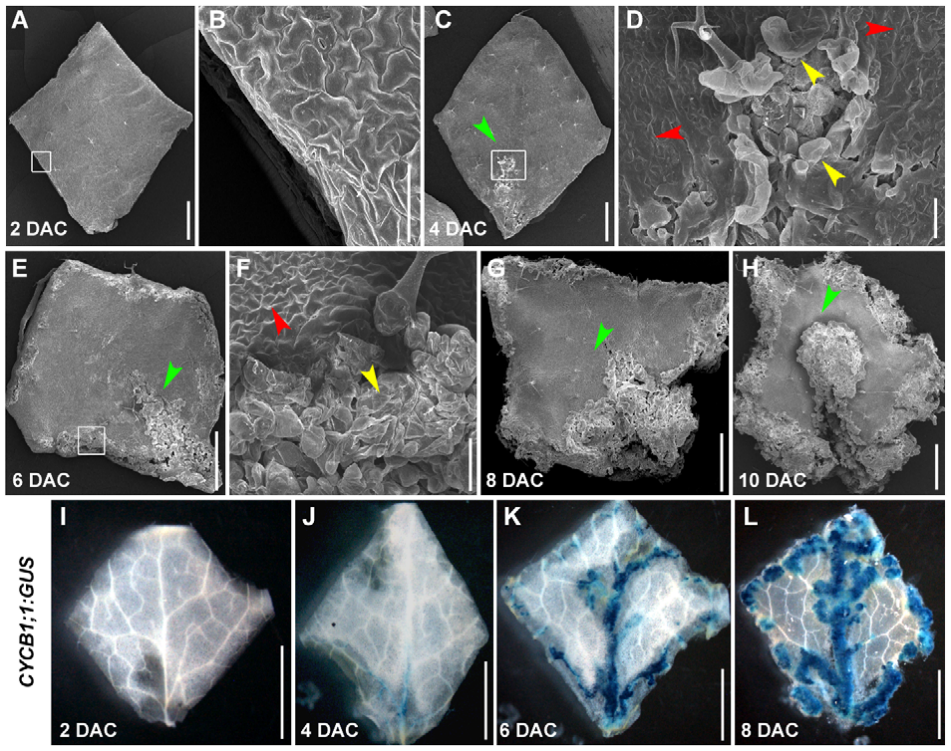
The leaf-to-callus transition process could be divided into pre-2 DAC and post-2 DAC stages based on the status of cell proliferation. (A–H) SEM analysis of morphology of leaf explants from 2 to 10 DAC, respectively. Green arrowheads indicate the midvein. Red arrowheads indicate leaf epidermal pavement cells. Yellow arrowheads show the dividing callus cells. Images in (B), (D), and (F) are close-ups of the boxed regions in (A), (C), and (E), respectively. (I–L) GUS staining of leaf explants from the *CYCB1;1:GUS* transgenic line from 2 to 8 DAC. Note that the 2 DAC explants show no GUS staining. Bars = 100 µm in (B, D, F), and 1 mm in (A, C, E, G, H) and 2 mm in (I–L).

To better understand callus development, we analyzed phenotypes of leaf explants during culturing using scanning electron microscopy (SEM). Shapes of leaf explants on CIM were unchanged 2 days after culturing (DAC) (Figure 3A), compared with explants prior to culturing (i.e., time 0) (Figure S1). Additionally, cell proliferation was not observed in the margin of 2 DAC explants (Figure 3A, 3B). In 4 DAC explants, cells started to proliferate, initially in the middle of the midvein (Figure 3C, 3D). Six days after culturing, cell proliferation occurred in the margin of leaf explants and vigorous cell division surrounding the midvein in the proximal part of the explants was observed (Figure 3E, 3F). Cell proliferation was clearly accelerated in 8 DAC and 10 DAC explants (Figure 3G, 3H), and the newly formed calli from the midvein and margins covered most parts of the 10 DAC explants (Figure 3H).

The *CYCB1;1* gene is strictly regulated by the cell cycle, and thus is widely used as a cell division marker [31]. By analyzing a *CYCB1;1:GUS* transgenic line, we demonstrated that the *CYCB1;1* expression pattern was consistent with the results from SEM. The 2 DAC leaf explants did not show any *CYCB1;1:GUS* staining (Figure 3I), whereas GUS staining was observed in the midvein and other fine veins in the proximal part of 4 DAC explants (Figure 3J). The strong *CYCB1;1:GUS* staining was observed along the midvein and vein branches, and in the margins of the 6 DAC and 8 DAC explants (Figure 3K, 3L). Based on the time of cell proliferation initiation, callus formation from leaf explants could be mainly divided into two stages: the pre- and post-2 DAC stages, reflecting prearrangement and performance of cell fate transition, respectively.

### H3K27me3 hypomethylation was first detected in the auxin response genes

**Figure 4:**
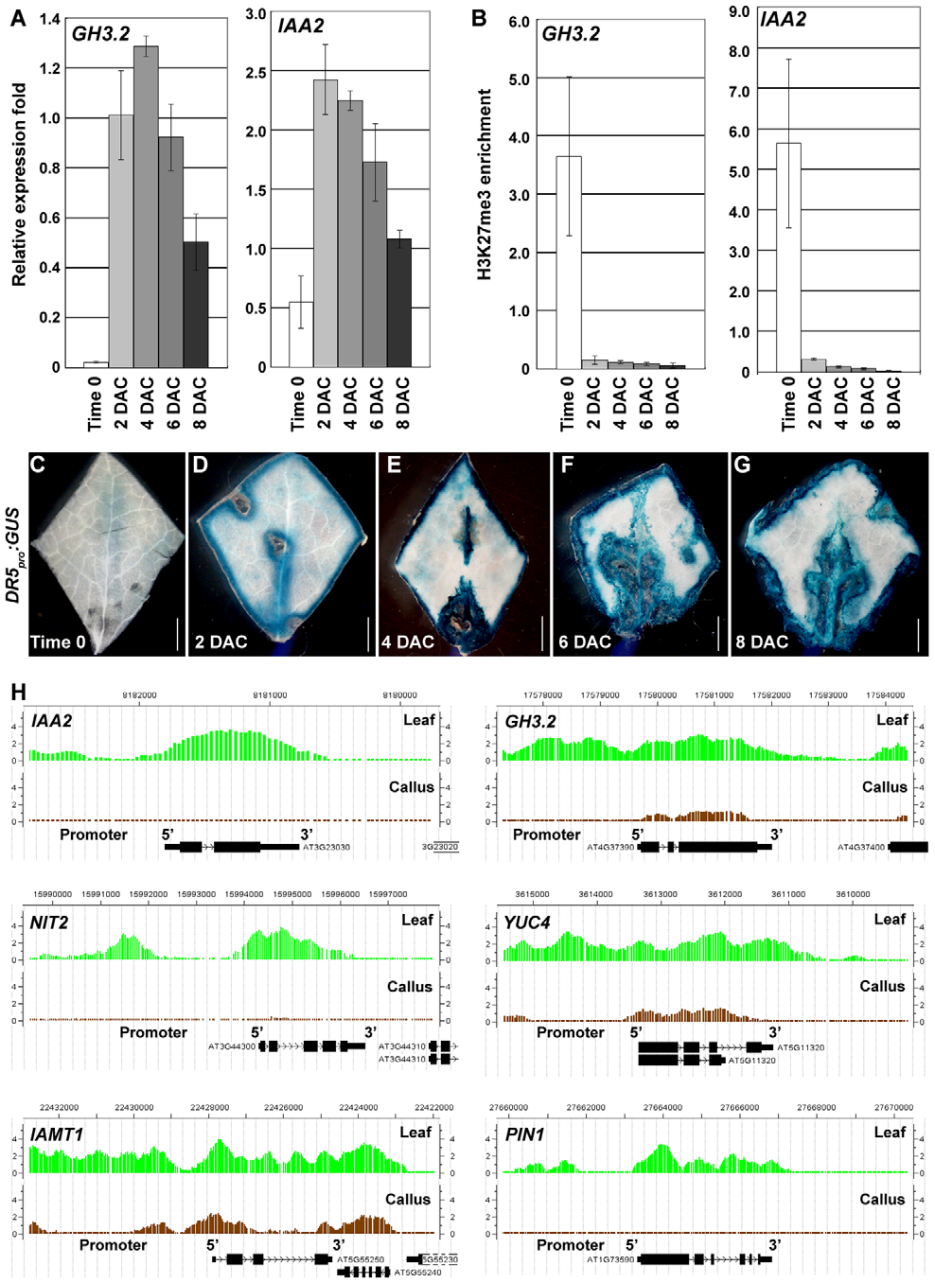
H3K27me3 hypomethylations at the auxin-pathway genes in the pre-2 DAC stage. (A) qRT-PCR analysis of *GH3.2* and *IAA2*. (B) ChIP analysis of *GH3.2* and *IAA2*. Bars show s.e. (C–G) GUS staining of leaf explants of the *DR5_pro_:GUS* transgenic line from time 0 to 8 DAC on CIM. (H) ChIP-chip results showed decreased H3K27me3 levels at *IAA2, GH3.2, NIT2*, *YUC4, IAMT1* and *PIN1* loci in calli, compared with those in leaves. Bars = 2 mm in (C–G).

Auxin is a plant hormone required for callus induction [2], and our ChIP-chip data revealed that H3K27me3 levels of a group of auxin-pathway genes were dramatically reduced in the callus compared with the leaf. These included several auxin-inducible genes, such as the *GH3* genes, *GH3.1*, *2*, *3*, *6*, and *17*, which are involved in auxin homeostatic control, and the *AUXIN/INDOLE-3-ACETIC ACID* (*AUX/IAA*) genes, *IAA1*, *2*, *14*, *19*, *20*, and *24* (Table S1), which participate in auxin signaling [32], [33]. Our qRT-PCR results revealed that these genes were induced before or on 2 DAC. For example, compared with the basal expression levels in time 0 explants, *GH3.2* expression levels were dramatically increased in 2 DAC explants and showed the highest levels in the 4 DAC explants (Figure 4A). The highest level of *IAA2* expression also appeared on 2 DAC (Figure 4A). We also analyzed the time course of the H3K27me3 level changes at the *GH3.2* and *IAA2* loci by ChIP (Figure 4B), and results were normalized against those at *AGAMOUS* (*AG*) because the H3K27me3 levels at the *AG* locus kept unchanged between the leaf and callus (Figure S2). Our results showed that increased expression levels of these auxin-pathway genes were accompanied by a sharp decrease in H3K27me3 levels at their loci starting from 2 DAC (Figure 4B). This suggested a dramatically altered auxin-related action in the pre-2 DAC explants.

The expression patterns of *GH3.2* and *IAA2* were consistent with those of the *DR5_pro_:GUS* reporter line [34], in which GUS staining was hardly detected at time 0 (Figure 4C), but was evident in the 2 DAC explants (Figure 4D). The strongest GUS staining was observed in 4 DAC explants (Figure 4E). Compared with the time 0 explants, which only showed very faint GUS signals surrounding the midvein (Figure 4C), deep GUS staining of the 2 DAC *DR5_pro_:GUS* explants was detected in the margins and midveins (Figure 4D). As cells were rapidly proliferating in 6 DAC and 8 DAC explants, all freshly formed calli were deeply stained (Figure 4F, 4G), indicating that a strong auxin signal transduction was occurring in these tissues.

In addition to the *GH3.2* and *IAA2* genes, other genes related to auxin biosynthesis, metabolism, and transport showed a significant loss in H3K27me3 (Figure 4H, Table S1). These genes included *YUCCA4* (*YUC4*) [35], *NITRILASE2* (*NIT2*) [36], *IAA CARBOXYL METHYLTRANSFERASE1* (*IAMT1*) [37], and *PIN-FORMED1* (*PIN1*) [38]. These results suggest that the entire network of auxin regulation is activated during the leaf-to-callus process.

### Changes in the H3K27me3 level in post-2 DAC explants may initiate cell fate transition

**Figure 5:**
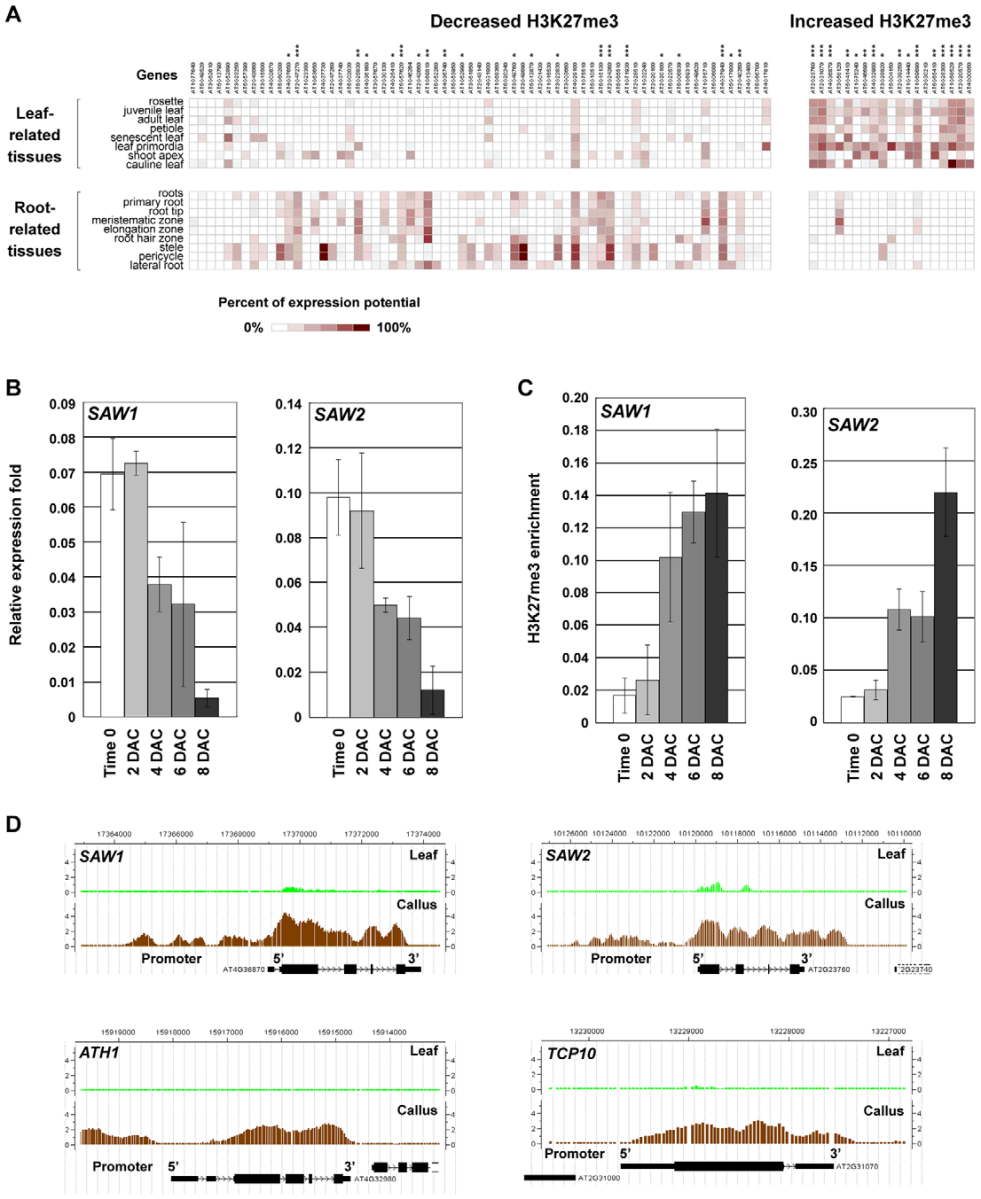
H3K27me3 reprogramming affects cell fate transition in the post-2 DAC stage. (A) The online program Genevestigator [30] was used to analyze expression patterns of the putative transcription factor genes that show changes in H3K27me3 levels during callus formation. Asterisks show significant statistical differences by t-tests (*, *p*<0.05; **, *p*<0.01; ***, *p*<0.001). Among the putative transcription factors with decreased H3K27me3 levels, 21 out of 67 (31.3%) genes have significantly higher expression patterns in the root tissues (left), and among those with increased H3K27me3 levels, 16 out of 19 (84.2%) genes have significantly higher expression patterns in the leaf tissues (right). (B) qRT-PCR analysis of *SAW1* and *SAW2*. (C) ChIP analysis of *SAW1* and *SAW2*. Bars show s.e. (D) ChIP-chip results in calli showed increased H3K27me3 levels at *SAW1, SAW2, ATH1*, and *TCP10*.

To understand the molecular basis during the leaf-to-callus transition, we analyzed expression patterns of all putative transcription factor genes that showed altered H3K27me3 levels in the ChIP-chip assay, as these genes may include important contributors to cell fate transition. The analysis was first performed using Genevestigator, which is a program available online (https://www.genevestigator.com/) [30]. Interestingly, many of these transcription factor genes with decreased H3K27me3 levels were those that were either silenced or exhibited low-level expression in the leaf, but were highly expressed in the roots (Figure 5A). Conversely, most genes that showed increased H3K27me3 levels were those that were originally predominantly expressed in the leaf, but were silenced or weakly expressed in the roots (Figure 5A). These results indicate that callus formation from leaf explants experiences a tissue identity transition from leaf to root, and thus support the recent proposal that the callus contains cells resembling the root meristematic pluripotent cells [5]. Our data also suggest that reprogramming of H3K27me3 is important for changes in expression of regulatory genes that are originally preferentially expressed in specific tissues.

We also analyzed gene expression levels over time by qRT-PCR and monitored H3K27me3 levels by ChIP for several selected marker genes of transcription factors during callus formation. *SAWTOOTH1* (*SAW1*) and *SAW2* are genes that control leaf development [39] and were selected as the leaf marker genes. Unlike the analyzed auxin-pathway genes, expression levels of *SAW1* and *SAW2* were largely unchanged 2 DAC, but were reduced markedly 4 DAC and continued to gradually decrease during the subsequent post-2 DAC stages (Figure 5B). The reduced *SAW1* and *SAW2* expression levels were accompanied by increased H3K27me3 levels beginning 4 DAC (Figure 5C, 5D). The increased H3K27me3 levels were observed for some other leaf development genes, such as the BELL family gene *ARABIDOPSIS THALIANA HOMEOBOX GENE1* (*ATH1*) [40] and the TEOSINTE BRANCHED1-CYCLOIDEA-PCF (TCP) transcription factor gene *TCP10* [41], [42] (Figure 5D, Table S1). These results suggest that PRC2-mediated H3K27me3 plays a role in silencing leaf regulatory genes during callus formation.

**Figure 6:**
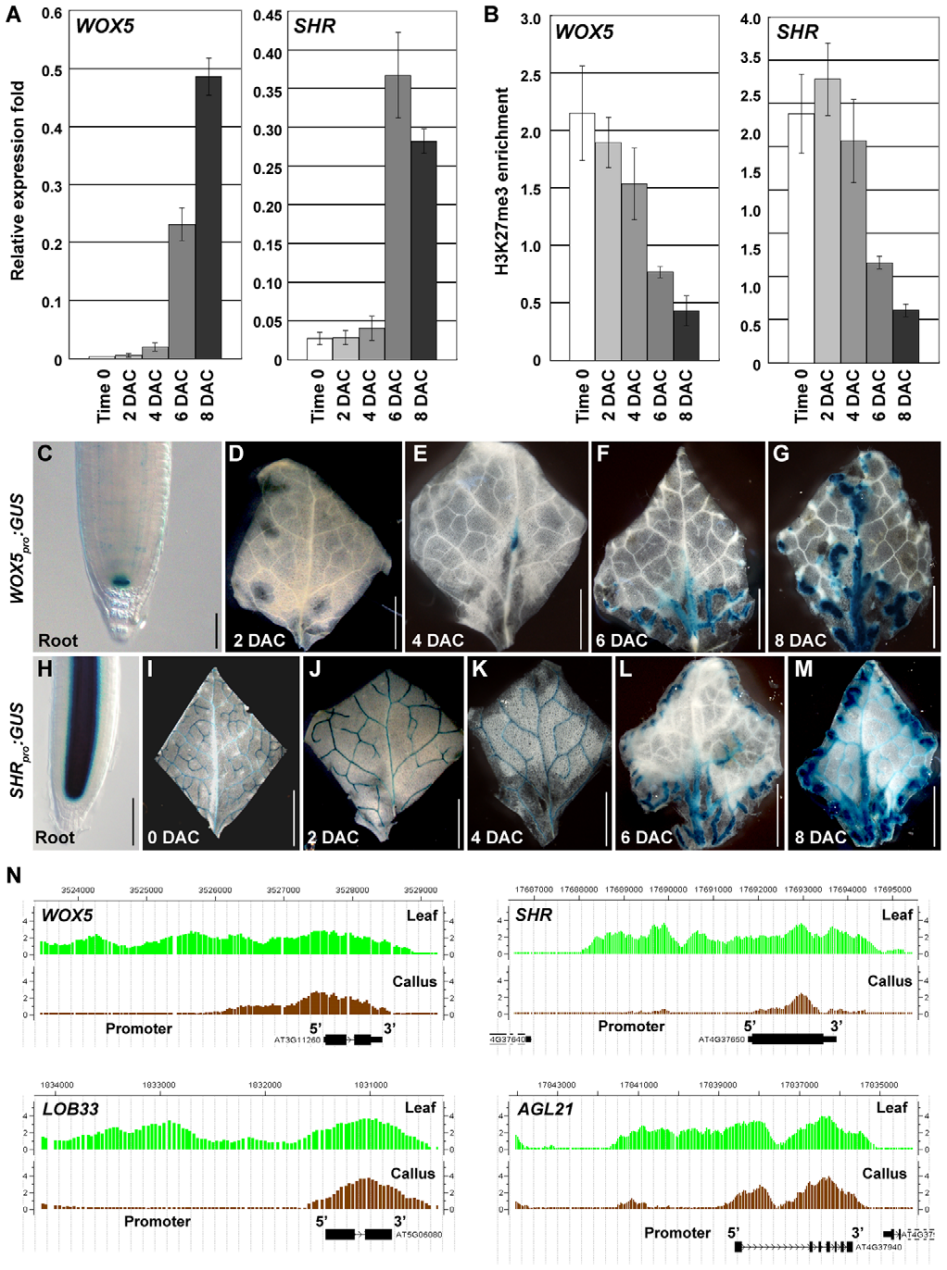
H3K27me3 hypomethylations occur at several root-regulatory genes. (A) qRT-PCR analyses show increased expression levels of root-regulatory genes *WOX5* and *SHR*. (B) ChIP analysis of *WOX5* and *SHR*. Bars show s.e. (C–G) GUS staining of the *WOX5_pro_:GUS* transgenic line in the root (C) and leaf explants cultured for 2 to 8 days on CIM (D–G). (H–M) GUS staining of the *SHR_pro_:GUS* transgenic line in the root (H), and in leaf explants at time 0 to 8 DAC (I–M). (N) ChIP-chip results in calli showed the reduced H3K27me3 levels at *WOX5, SHR, LOB33* and *AGL21*. Bars = 100 µm in (C, H) and 2 mm in (D–G, I–M).

*WUSCHEL-RELATED HOMEOBOX 5* (*WOX5*) is a root gene specifically expressed in the quiescent center (QC) and *SHORT-ROOT* (*SHR*) is an important root development-controlling gene [43], [44]. *WOX5* was slightly derepressed in the 4 DAC explants and was highly expressed in 6 DAC and 8 DAC explants. A drastic *SHR* derepression also occurred 6 DAC (Figure 6A). The increased *WOX5* and *SHR* expression levels were consistent with the decreased H3K27me3 levels starting 4 DAC (Figure 6B), suggesting that H3K27 demethylation is also required for derepression of root genes.

We then used *WOX5_pro_:GUS* and *SHR_pro_:GUS* transgenic plants to analyze their expression patterns during callus formation. In the *WOX5_pro_:GUS* transgenic line, GUS staining was only detected in the root QC cells (Figure 6C) [43]. *WOX5* remained silenced in leaf explants 2 DAC (Figure 6D), but expression in the midvein was observed 4 DAC (Figure 6E). GUS staining in 6 DAC and 8 DAC explants was observed in the midvein and the fine vein ends close to the wounded margins in emerging calli. This staining became more intense with increasing cell proliferation (Figure 6F, 6G). These results indicate that many cells in the newly formed calli from leaf explants possess features of root QC cells.

*SHR* is also expressed in the leaf vascular system (Figure 6I) in addition to its expression in the root stele cells (Figure 6H) [44]. However, *SHR* expression in early-stage explants is obviously different from that in the late-stage explants. The *SHR* expression pattern in the leaf vein did not change from time 0 to 4 DAC (Figure 6I–6K). In the 6 DAC leaf explants, *SHR* expression was weakened in the original leaf veins but became more intense in the midvein and vein ends near the wounded margin of newly formed calli (Figure 6L, 6M). In addition to *WOX5* and *SHR*, the H3K27me3 levels of some other root genes, such as *LATERAL ORGAN BOUNDARY DOMAIN33* (*LOB33*) [45] and *AGAMOUS-LIKE21* (*AGL21*) [46], were also consistently decreased in the post-2 DAC stage (Figure 6N, Table S1). Changes in expression and H3K27me3 levels of the analyzed leaf and root regulatory genes highlight the process of cell fate transition occurring during callus formation in the post-2 DAC stage, following the auxin-pathway activation in the pre-2 DAC stage.

### PcG plays a major role in the elimination of leaf characteristics during callus formation

**Figure 7:**
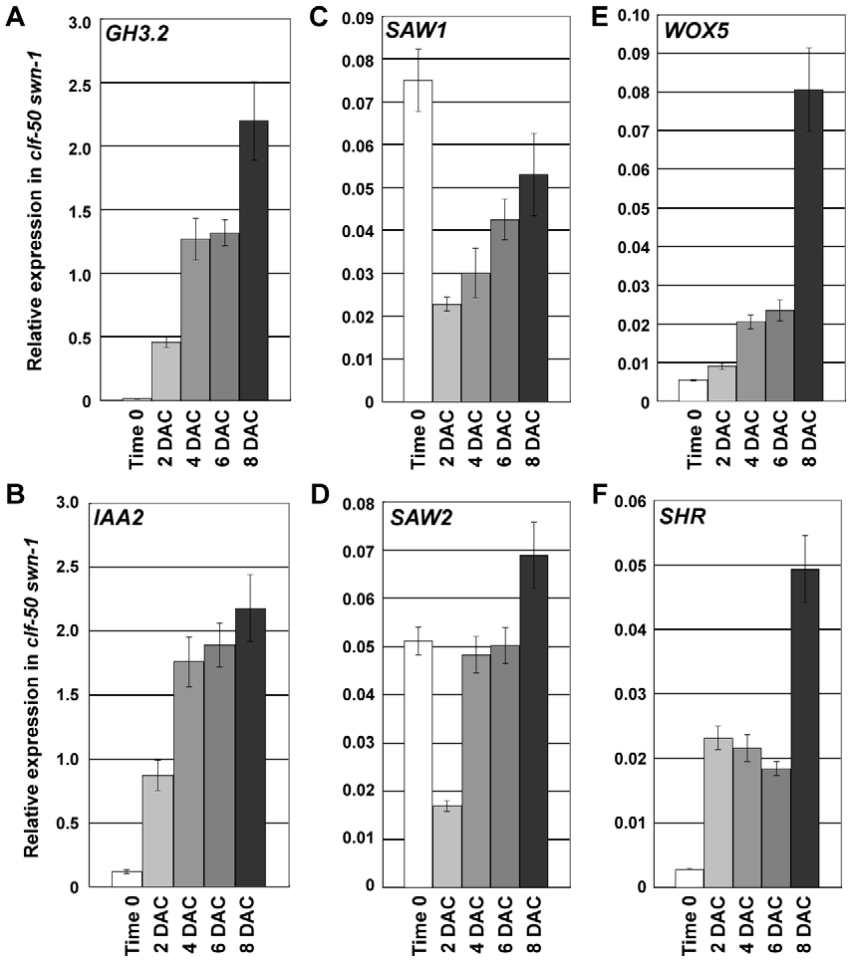
PcG is required for silencing leaf-regulatory genes during the leaf-to-callus transition. (A–F) Expression level changes of *GH3.2* (A), *IAA2* (B), *SAW1* (C), *SAW2* (D), *WOX5* (E), and *SHR* (F) in leaf explants of *clf-50 swn-1* from time 0 to 8 DAC, revealed by qRT-PCR analyses. Bars show s.e.

To determine the role that the PRC2 plays in callus formation, we analyzed the expression of selected regulatory genes in the *clf-50 swn-1* mutant. Both *GH3.2* and *IAA2* showed a similar elevated expression compared to the wild-type counterparts, suggesting that auxin response may not involve PRC2 (Figure 7A, 7B). Expression levels of *SAW1* and *SAW2* were reduced (Figure 7C, 7D) and those of *WOX5* and *SHR* were elevated (Figure 7E, 7F) in 2 DAC leaf explants of *clf-50 swn-1*, rather than the expression changes occurring in 4 DAC leaf explants in the wild type. This was indicative of a shortened pre-2 DAC stage in the PRC2 mutant. Interestingly, expression levels of *SAW1* and *SAW2* in *clf-50 swn-1* leaf explants failed to continue to decrease during the post-2 DAC stage (Figure 7C, 7D), as was observed in the wild type (Figure 5B), but instead gradually increased. On the other hand, the H3K27me3 modification at the *SAW1* and *SAW2* loci consistently retained at a very low level in *clf-50 swn-1* (Figure S3). These results indicate that the leaf explants of *clf-50 swn-1* consistently retain their leaf identities, although *WOX5* and *SHR* were derepressed during culturing (Figure 7E, 7F).

**Figure 8:**
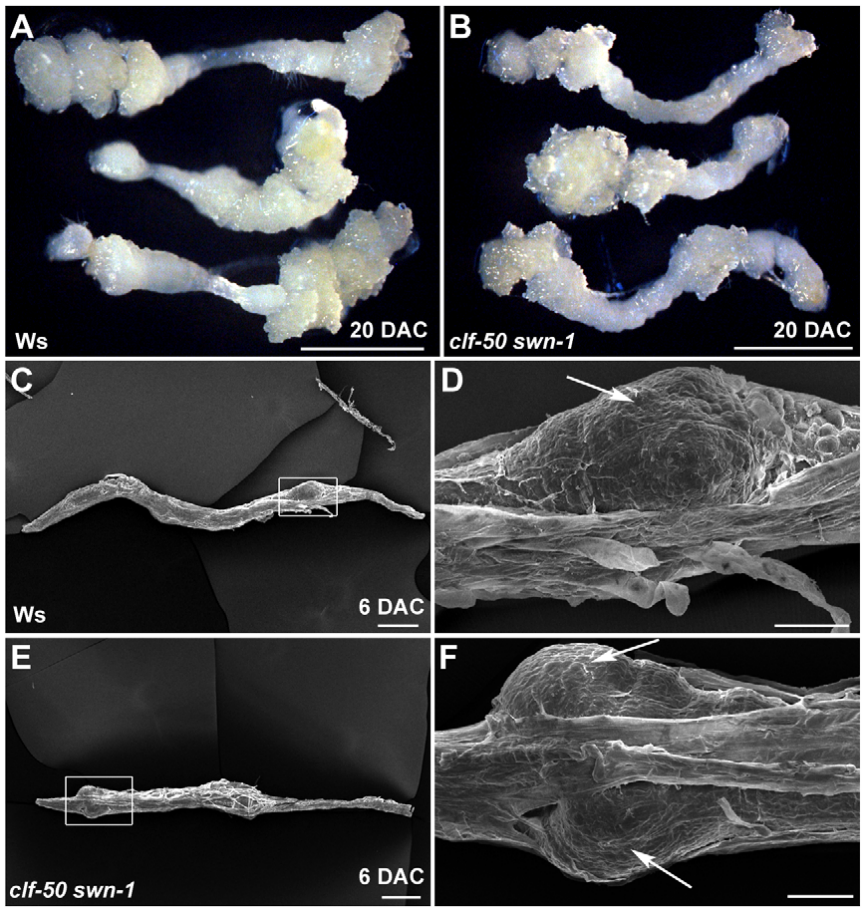
The root explants of the PcG mutant can form a callus normally. (A, B) Calli of both 20 DAC root explants of wild-type Ws (A) and *clf-50 swn-1* (B) are both evident. (C–F) SEM analyses showed that calli on root explants of both Ws (C, D) and *clf-50 swn-1* (E, F) 6 DAC are of similar sizes. (D) and (F) are close-ups of the boxed regions in (C) and (E), respectively. Arrows indicate the newly formed calli. Bars = 2 mm in (A, B), 200 µm in (C, E), and 50 µm in (D, F).

Because PcG is required for silencing of the leaf development-controlling genes, but does not affect the derepression of auxin- and root-related genes, it is possible that leaf and root explants of the PRC2 mutant might behave differently during callus formation. To test this hypothesis, we analyzed the ability of the *clf-50 swn-1* roots to form calli. Similar to the wild type roots (Figure 8A), the *clf-50 swn-1* root explants demonstrated normal callus formation (Figure 8B). A more detailed analysis using SEM showed that the progression of callus formation in wild type (Figure 8C, 8D) and *clf-50 swn-1* (Figure 8E, 8F) was also similar, as callus cells were evident on both root explants 6 DAC. These results indicate that PcG is required for the leaf, but not the root, during callus formation.

## Discussion

Recent studies have revealed that callus formation occurs through a lateral root development pathway [5]. In this study, we tried to address another layer of questions during callus formation: what is the molecular mechanism that guides different plant tissues to form the pluripotent root meristem-like cells. We show that PcG-mediated epigenetic regulation is an essential step in callus formation, and the PRC2 components CLF and SWN are required for the leaf-to-callus transition. In addition to the rosette leaves, we analyzed cotyledons of *clf-50 swn-1*, and they were also defective in forming callus (Figure S4). To determine whether PRC2 as a whole is required for cell fate changes, we analyzed another PRC2 component EMF2 [21], [23] by examining the regeneration ability of *emf2* cotyledons, because of a lack of leaves in *emf2*. Similar to those of *clf-50 swn-1*, cotyledons from two different *emf2* alleles, *emf2-1* and *emf2-11*, were both defective in callus formation (Figure S4), indicating the importance of PRC2 complex in the leaf-to-callus transition.

Our ChIP-chip assay revealed that reprogramming of H3K27me3 occurred at several important transcription factor genes, which regulate plant meristematic functions. For example, *SAW1*, *SAW2*, and *TCP* genes are known to control the leaf margin by repression of leaf meristematic features [39], [42], and *WOX*, *KNOX*, and several *BELL* family genes are also known to be involved in root or shoot meristematic activities (Table S1) [47], [48]. It is possible that maintenance of the undifferentiated state of cells requires functions of these genes. In addition, a number of metabolism-related genes showed notable changes in the H3K27me3 level, probably reflecting a secondary effect during changes of cell fates. Interestingly, transposable elements are generally much less regulated by PRC2-mediated H3K27me3 [13], whereas H3K27me3 levels at many transposable elements were elevated during callus formation (Figure 2C; Table S1). It is of interest to test in the future whether reprogramming of H3K27me3 at the loci of these transposable elements contributes to the callus formation.

The PcG pathway plays an important role in the leaf-to-callus transition; however, since PcG regulates many target genes during this process, the exact molecular mechanism still remains elusive. The fact that many leaf genes fail to be repressed in leaf explants of the *clf-50 swn-1* double mutant could be one possible reason for the defective callus formation from leaf blades, and several lines of evidence support this hypothesis.

First, during callus formation from certain aerial organs of a plant, the differentiated tissues must undergo a common process to eliminate their original characteristics [5]. PcG has long been known to function in switching cell features not only in plants but also in animals. Second, most genes with increased H3K27me3 levels during callus formation are those that are highly expressed in the leaf but weakly in the root, including *SAW1*, *SAW2* and many others (Figure S5). Consistent with *SAW1* and *SAW2*, these genes demonstrated insufficient repression in leaf explants of *clf-50 swn-1* (Figure S5). Third, similar to those of wild type, leaf explants of *clf-50 swn-1* also show a gradually increased root gene expression, such as the demonstrated *WOX5* and *SHR* genes, and their expression levels between wild-type and *clf-50 swn-1* leaf explants are also similar within 4 DAC (Figure 6A, 7E and 7F). Although after 6 DAC, expression levels of these root genes became much higher in wild type than in the double mutant, this dramatic increase of expression levels might result from the newly formed calli but not from the leaf explants, as the *clf-50 swn-1* explants failed to form callus. Thus, the *clf-50 swn-1* mutant has the same potential to express the root genes but is unable to silence the leaf genes to suspend the leaf program in the leaf explants. The obstacle that must be overcome for callus formation in the leaf explants does not exist in the root explants of *clf-50 swn-1* and *emf2*, providing a possible explanation why *clf-50 swn-1* and *emf2* root explants possess the ability to form calli (Figure 8B; Figure S4). Although the elimination of leaf characteristics appears to be a necessary step for callus formation from leaf explants, we could not exclude the possibility that this process is controlled by some other not-yet-revealed mechanisms.

To identify key genes that preferentially expressed in the leaf and may contribute to the leaf characteristic, we overexpressed *ATH1* and *SAW1* and analyzed callus formation of leaf explants from *35S_pro_:ATH1* and *35S_pro_:SAW1* transgenic plants. However, overexpression of the genes did not affect the leaf-to-callus transition (Figure S6). It is possible that for maintenance of leaf characteristics, many other leaf-expressed genes may be equally important.

The *clf-50 swn-1* double mutant flowers earlier [21], indicating a shortened vegetative growth phase in *clf-50 swn-1*. This phenotype of *clf-50 swn-1* raises a question: whether the *clf-50 swn-1* leaves in ages are all equivalent to the very old wild-type ones that may lose the ability to form calli. We thus tested callus formation using wild-type and *clf-50 swn-1* tissues at different ages, and found that cotyledons and leaf blades from the 44-day-old Col-0 seedlings were able to form calli, whereas the young 9-day-old leaf blades of the *clf-50 swn-1* seedlings were defective in callus formation (Figure S6). These results suggest that the defective PcG function but not the age of tissues in *clf-50 swn-1* blocked the callus formation.

During leaf-to-callus transition, the CLF and SWN proteins may act redundantly in regulating downstream genes, as the leaf explants of both *clf-50* and the stronger *swn-21* single mutants normally form calli (Figure S7). The weak *clf-50 swn-1* double mutant usually produces only four rosette leaves. We tested each of these leaves at different leaf developmental stages and found the phenotype of defective regeneration was very consistent (Figure 1F; Figure S7).

Similar to rosette leaves, cotyledons of *clf-50 swn-1*, *emf2-1* and *emf2-11* are also defective in callus formation (Figure S4). Although rosette leaves and cotyledons are different plant organs, they may partially share a common program, which must be terminated by PcG for regeneration. Whether the PRC2-mediated H3K27me3 is responsible for initiating or maintaining leaf gene silencing during the leaf-to-callus transition is not yet clear. It has recently been suggested that in both animals and plants, histone modifications might not initiate gene expression regulation [12], [17], [49]. In *Arabidopsis*, it was found that H3K27me3 alone is not sufficient to initiate the repression of the *AG* gene [12], [17]. Our data showed that the leaf genes, *SAW1* and *SAW2*, were downregulated in the 2 DAC leaf explants of *clf-50 swn-1*, whereas such a downregulation could not be maintained at or following 4 DAC. One possible explanation for this is that the PRC2-mediated H3K27me3 is responsible for maintaining rather than initiating the silencing of leaf genes.

A leaf-to-callus transition normally requires both repression of leaf-regulatory genes and activation of auxin-pathway and root-regulatory genes. The PcG-mediated histone modification and the H3K27 demethylation pathways may act in parallel in reprogramming of H3K27me3 during callus formation. How the pre- and post-2 DAC stages are connected is not known. Whether the activated auxin pathway in the pre-2 DAC stage induces a subsequent reprogramming of H3K27me3 at the loci of the leaf- and root-regulatory genes is an interesting question that will be addressed in the future.

Removal of the H3K27me3 marker may depend on an active process involving H3K27 demethyltransferases, such as REF6 [24], or a passive process resulting in the loss of the maintenance of histone methylations during cell division [50]. We propose that in the pre-2 DAC stage, H3K27me3 demethylation of the auxin-pathway genes is mainly via an active process because during this stage, cell division does not occur. However, during the post-2 DAC stage when cells start to proliferate, H3K27 demethylation leading to derepression of the root-regulatory genes may be via both active and passive processes. We tried to address this question by analyzing leaf explants of the *ref6-1* mutant, but found that normal *ref6-1* calli formed from leaf explants (Figure S7). These results suggest that *REF6* may not be the only demethyltransferase in callus formation or may not participate in callus formation at all, and/or passive removal of H3K27 methylation may occur. Elucidation of the molecular mechanism of H3K27me3 demethylation during callus formation is important to fully comprehend plant cell regeneration.

In animals, cell fate determinations also require the PcG function for acquisition of cell pluripotency [51], [52]. In plants, determination of flowering time, floral organ formation, and stem cell restriction are all related to reprogramming of H3K27me3 and require PcG [17], [19], [21], [53]–[55]. Reprogramming of H3K27me3 might represent a general mechanism for cell fate transition in multicellular eukaryotes and the PcG pathway is at least partially involved in the process.

## Materials and Methods

### Plant materials and callus induction

Seeds of *clf-50 swn-1* is in the Ws background, and *swn-1* is a weak *swn* allele [21]. Seeds of *sdg2-3* [26], *sdg8-2* [28], *atx1-2* [56] and *ref6-1* [57], *swn-21* (GK-783A01), *emf2-11* (GK-685A01) [58], *emf2-1* [59], *clf-29* [60], and the *CYCB1;1:GUS* [31] and *DR5_pro_:GUS* [34] transgenic plants are in the Col-0 background. For callus induction, seeds were first grown on the medium containing Murashige and Skoog (MS) basal salt mixture. Tissues from different organs were prepared from different ages of seedlings and were placed on CIM (Gamborg's B5 medium with 0.5 g/L MES, 2% glucose, 0.2 mmol/L kinetin, and 2.2 mmol/L 2,4-dichlorophenoxyacetic acid and 0.8% agar) [61], followed by incubation at 22°C in the dark.

### β-glucuronidase (GUS) assay and microscopy

DNA fragments with 4.6 kb and 2.5 kb in length upstream to the translation initiation site of *WOX5* and *SHR* were PCR amplified from wild-type plants, respectively, and were subcloned into the plant transformation vector pBI101 (Clonetech, USA) with the 3′ in-frame fusion to GUS to yield *WOX5_pro_:GUS* and *SHR_pro_:GUS*, respectively. The constructs were verified by sequencing and were introduced into wild-type plants by *Agrobacterium*-mediated transformation. Primers used in cloning are shown in Table S3. For GUS staining, plant tissues were incubated in the GUS assay solution (50 mM sodium phosphate buffer pH 7, 5 mM Na_2_EDTA, 2 mM K_3_Fe(CN)_6_, 2 mM K_4_Fe(CN)_6_, 0.1% Triton X-100 and 0.04% X-Gluc) at 37°C for 90 min for the *DR5_pro_:GUS* or for 3 h for the *CYCB1;1:GUS*, *WOX5_pro_:GUS*, and *SHR_pro_:GUS* leaf explants, and the stained tissues were then incubated in 70% alcohol at 37°C for 12 h. GUS staining was observed by using the SMZ1500 and Eclipse 80i microscopes (Nikon, Japan). SEM analysis was performed as previously described [62].

### ChIP and qRT–PCR

For the Chromatin immunoprecipitation (ChIP) assay, leaf explants cultured at different time points on CIM or the 20 DAC calli were vacuum-infiltrated with formaldehyde crosslinking solution. ChIP was performed as previously described [29], by using the antibody against H3 trimethyl-Lys 27 (Upstate, USA, Cat. 07-449). Results from real-time PCR represented the relative methylation levels, which were normalized against those produced by the primers for *AG* (Figure S2), whose values were arbitrarily fixed at 1.0.

For quantitative reverse transcription-polymerase chain reaction (qRT-PCR), RNA extraction and reverse transcription were performed as described previously [62], [63]. Three biological replicates were analyzed and each was tested by three technical replicates. Real-time PCR was performed using gene specific primers (Table S3) and the results represented the relative expression levels, which were normalized against those produced by the primers for *ACTIN*, whose values were arbitrarily fixed at 1.0.

### Microarray, ChIP–chip assay, and data analysis

For microarray analysis, leaves from 20-day-old seedlings of wild type and 20 DAC calli were used for RNA preparation. Microarray was performed using the Affymetrix GeneChip system (Affymetrix, USA, and Gene Tech Biotechnology Company Limited, Shanghai, China). Three independent experiments were performed, and expression of genes was considered significantly perturbed when the change was more than 2.0-fold with a P value<0.05. The microarray data were deposited in the Gene Expression Omnibus (GEO, http://www.ncbi.nlm.nih.gov/geo/) under the accession numbers GSE36479 and the analyzed data were shown in Table S2.

For ChIP-chip, about 100 ng DNA from leaves or calli was enriched, respectively, in the ChIP assay, and the ChIP-chip experiment was carried out by using Affymetrix high density tiling arrays according to the manufacturer's protocol (Cat. 900594, Affymetrix, USA, and Gene Tech Biotechnology Company Limited, Shanghai, China). ChIP-chip data were analyzed using the CisGenome software with standard procedures [64], [65]. Genes with the significantly decreased H3K27me3 levels were defined as the peak cutoff MA (Leaf-Callus)≥3.5 and MA (leaf)≥2.5, and those with the significant increased H3K27me3 levels were defined as MA (Callus-Leaf)≥3.5 and MA (Callus)≥2.5. FDR (<5%) was settled as “Left ta”, which was recommended by the CisGenome software. The peak-gene association was settled from 3,000 bp upstream to the transcription start site (TSS) to the transcription end site (TES). The database TAIR8 was used for annotation of the identified genes. For genes cluster based on the tissue specific expression, an online program Genevestigator (www.genevestigator.com) [30] was used. The ChIP-chip data were deposited in the GEO (http://www.ncbi.nlm.nih.gov/geo/) under the accession numbers GSE34596 and the analyzed data are shown in Table S1.

## Supporting Information

Figure S1 SEM analysis of the time 0 leaf explant. (A) A time 0 leaf explant derived from the rosette leaf of the 20-day-old wild-type Col-0 plant. (B) Close-up of the boxed region in (A). Bars = 1 mm in (A) and 50 µm in (B).
(TIF)

Figure S2 H3K27me3 modifications in the *AG* locus. Analyses of the *AG* locus reveal that the H3K27me3 patterns and levels are very similar between leaves and calli.
(TIF)

Figure S3 ChIP assays at different timepoints to analyze H3K27me3 levels of the *SAW1* and *SAW2* loci in the *clf-50 swn-1* mutant. (A) The time 0 and 8 DAC leaf explants of the *clf-50 swn-1* double mutant. (B) The 20-day-old roots of the wild-type Ws and the *clf-50 swn-1* mutant plants. Values of 1% input from time-0 leaf explants (A) or the *clf-50 swn-1* roots (B) were arbitrarily fixed at 1.0. Note that the H3K27me3 modification was at a very low level at the *SAW1* and *SAW2* loci in both Ws and *clf-50 swn-1* roots. These results suggest that the PcG function is required for repression of leaf genes during the leaf-to-callus transition, whereas root tissues may bypass the process of leaf feature elimination, such that their callus formation does not require the PcG function.
(TIF)

Figure S4 Cotyledons of *clf-50 swn-1*, *emf2-11* and *emf2-1* are defective in callus formation. (A–E) Cotyledon explants of wild-type Ws (A), wild-type Col-0 (B), *clf-50 swn-1* (C), *emf2-11* (D) and *emf2-1* (E). Nine-day-old cotyledons were cut at the junction between the blade and the petiole and the blade parts were cultured on CIM for another 20 days. For each genotype, 10 cotyledon explants were tested for regeneration. While all cotyledon explants from the wild types formed calli, all those from the mutants, except two for *clf-50 swn-1* (C, one is shown in right panel) and one for *emf2-1* (E, right panel), showed the complete block in regeneration. (F and G) Root explants of *emf2-11* (F) and *emf2-1* (G) formed calli. Bars = 2 mm in (A, B) and 1 mm in (C–G).
(TIF)

Figure S5 Insufficient repression of the leaf-preferentially expressed regulatory genes in the *clf-50 swn-1* double mutant. (A) A total of 19 leaf-preferentially expressed putative transcription factor genes were analyzed, and results from 18 genes were obtained and all showed an insufficient repression pattern in *clf-50 swn-1* as compared with that in the wild type. (B–F) Expression patterns of 5 genes, *ATHB34* (AT3G28920) (B), *AtTCP15* (AT1G69690) (C), *BEL1* (AT5G41410) (D), *ATH1* (AT4G32980) (E), and *TCP10* (AT2G31070) (F), are shown. qRT-PCR was performed using time 0 to 8 DAC leaf explants. Bars show s.e.
(TIF)

Figure S6 Callus induction using tissues from wild-type, *clf-50 swn-1*, *35S_pro_:SAW1*, and *35S_pro_:ATH1* plants. (A and B) Cotyledons (A) and leaf blades (B) from 44-day-old Col-0 seedlings. (C and D) Leaf blades from 9-day-old (C) and roots from 30-day-old (D) *clf-50 swn-1* seedlings. (E and F) Leaf blades from 15-day-old *35S_pro_:SAW1*/Col-0 (E) and *35S_pro_:ATH1*/Col-0 (F) seedlings. For each test, 20 leaf blades or cotyledons were cultured, and all explants exhibited consistent phenotypes, except one *clf-50 swn-1* leaf blade formed callus. *35S_pro_:SAW1* and *35S_pro_:ATH1* were constructed by cloning the cDNAs encoding the full length proteins driven by the *35S* promoter. Bars = 1 mm in (A–C, E and F) and 1 mm in (D).
(TIF)

Figure S7 Regeneration abilities of leaf explants of *clf-50 swn-1*, *clf-50*, *swn-21*, and *ref6-1*. (A) The first two 20-day-old rosette leaves of *clf-50 swn-1*. (B–D) The third and fourth 20-day-old rosette leaves of *clf-50* (B), *swn-21* (C) and *ref6-1* (D). More than 30 blades were tested for each mutant, and they all exhibited the consistent phenotype. Bars = 1 mm in (A) and 2 mm in (B–D).
(TIF)

Table S1 ChIP-chip data.
(XLS)

Table S2 Microarray data.
(XLS)

Table S3 List of primers used in this study.
(DOC)
