# Supplementary figures and images for: Reprogramming of H3K27me3 Is Critical for Acquisition of Pluripotency from Cultured Arabidopsis Tissues
Source: PLoS Genet. 2012 Aug 23;8(8):e1002911. doi: 10.1371/journal.pgen.1002911 (PMC3426549; doi:10.1371/journal.pgen.1002911)

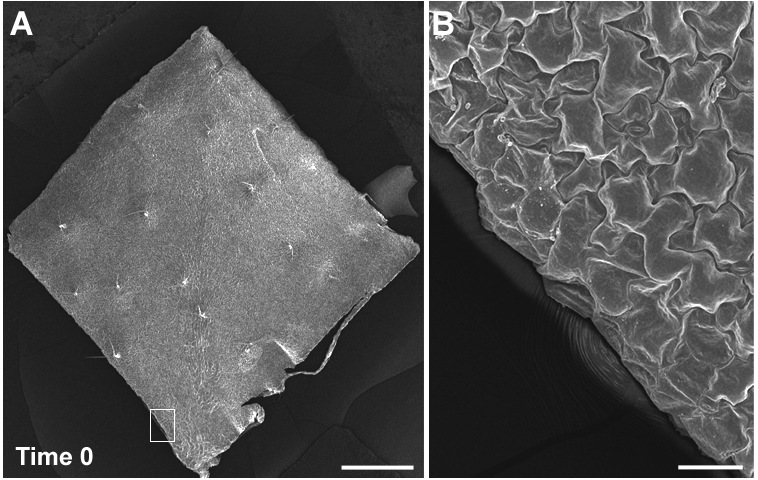

Supplement: Figure S1 — SEM analysis of the time 0 leaf explant. (A) A time 0 leaf explant derived from the rosette leaf of the 20-day-old wild-type Col-0 plant. (B) Close-up of the boxed region in (A). Bars = 1 mm in (A) and 50 µm in (B). (TIF) [file pgen.1002911.s001.tif]

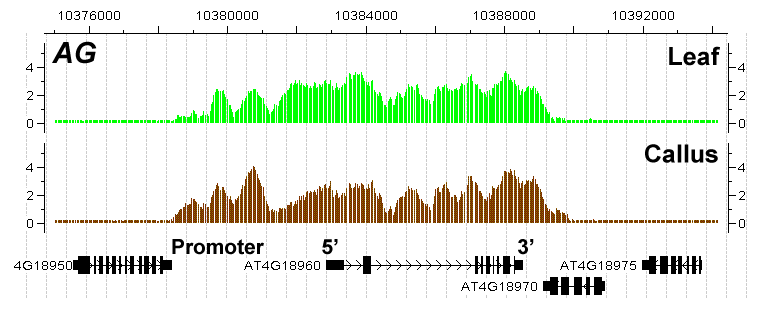

Supplement: Figure S2 — H3K27me3 modifications in the AG locus. Analyses of the AG locus reveal that the H3K27me3 patterns and levels are very similar between leaves and calli. (TIF) [file pgen.1002911.s002.tif]

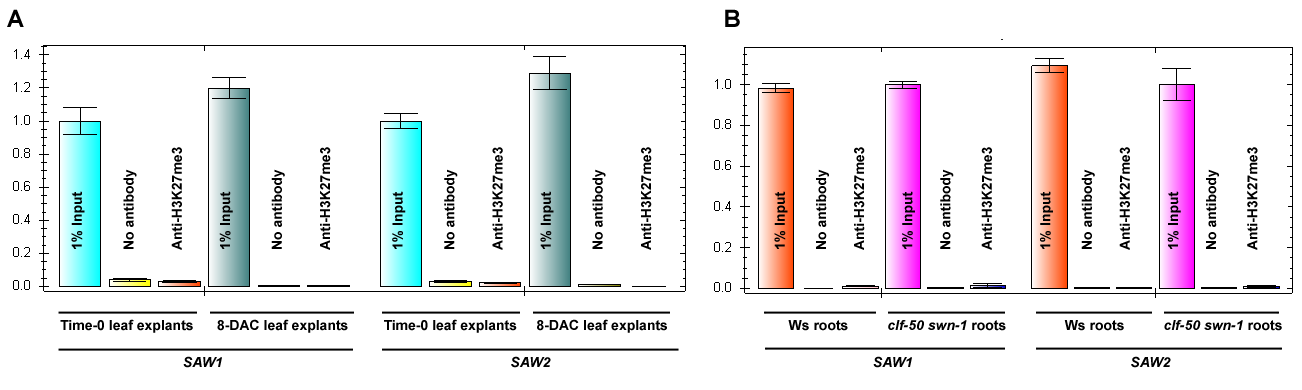

Supplement: Figure S3 — ChIP assays at different timepoints to analyze H3K27me3 levels of the SAW1 and SAW2 loci in the clf-50 swn-1 mutant. (A) The time 0 and 8 DAC leaf explants of the clf-50 swn-1 double mutant. (B) The 20-day-old roots of the wild-type Ws and the clf-50 swn-1 mutant plants. Values of 1% input from time-0 leaf explants (A) or the clf-50 swn-1 roots (B) were arbitrarily fixed at 1.0. Note that the H3K27me3 modification was at a very low level at the SAW1 and SAW2 loci in both Ws and clf-50 swn-1 roots. These results suggest that the PcG function is required for repression of leaf genes during the leaf-to-callus transition, whereas root tissues may bypass the process of leaf feature elimination, such that their callus formation does not require the PcG function. (TIF) [file pgen.1002911.s003.tif]

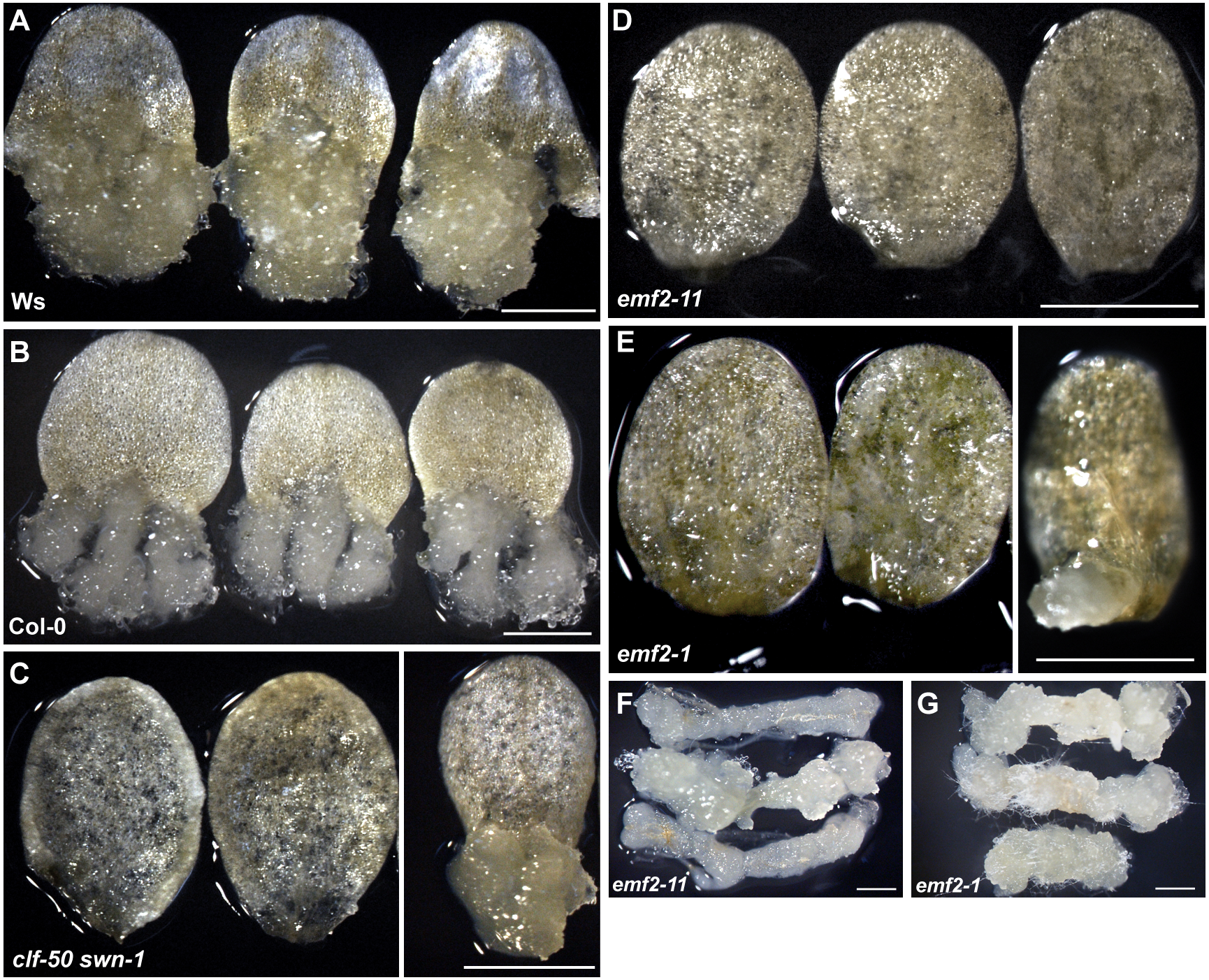

Supplement: Figure S4 — Cotyledons of clf-50 swn-1, emf2-11 and emf2-1 are defective in callus formation. (A–E) Cotyledon explants of wild-type Ws (A), wild-type Col-0 (B), clf-50 swn-1 (C), emf2-11 (D) and emf2-1 (E). Nine-day-old cotyledons were cut at the junction between the blade and the petiole and the blade parts were cultured on CIM for another 20 days. For each genotype, 10 cotyledon explants were tested for regeneration. While all cotyledon explants from the wild types formed calli, all those from the mutants, except two for clf-50 swn-1 (C, one is shown in right panel) and one for emf2-1 (E, right panel), showed the complete block in regeneration. (F and G) Root explants of emf2-11 (F) and emf2-1 (G) formed calli. Bars = 2 mm in (A, B) and 1 mm in (C–G). (TIF) [file pgen.1002911.s004.tif]

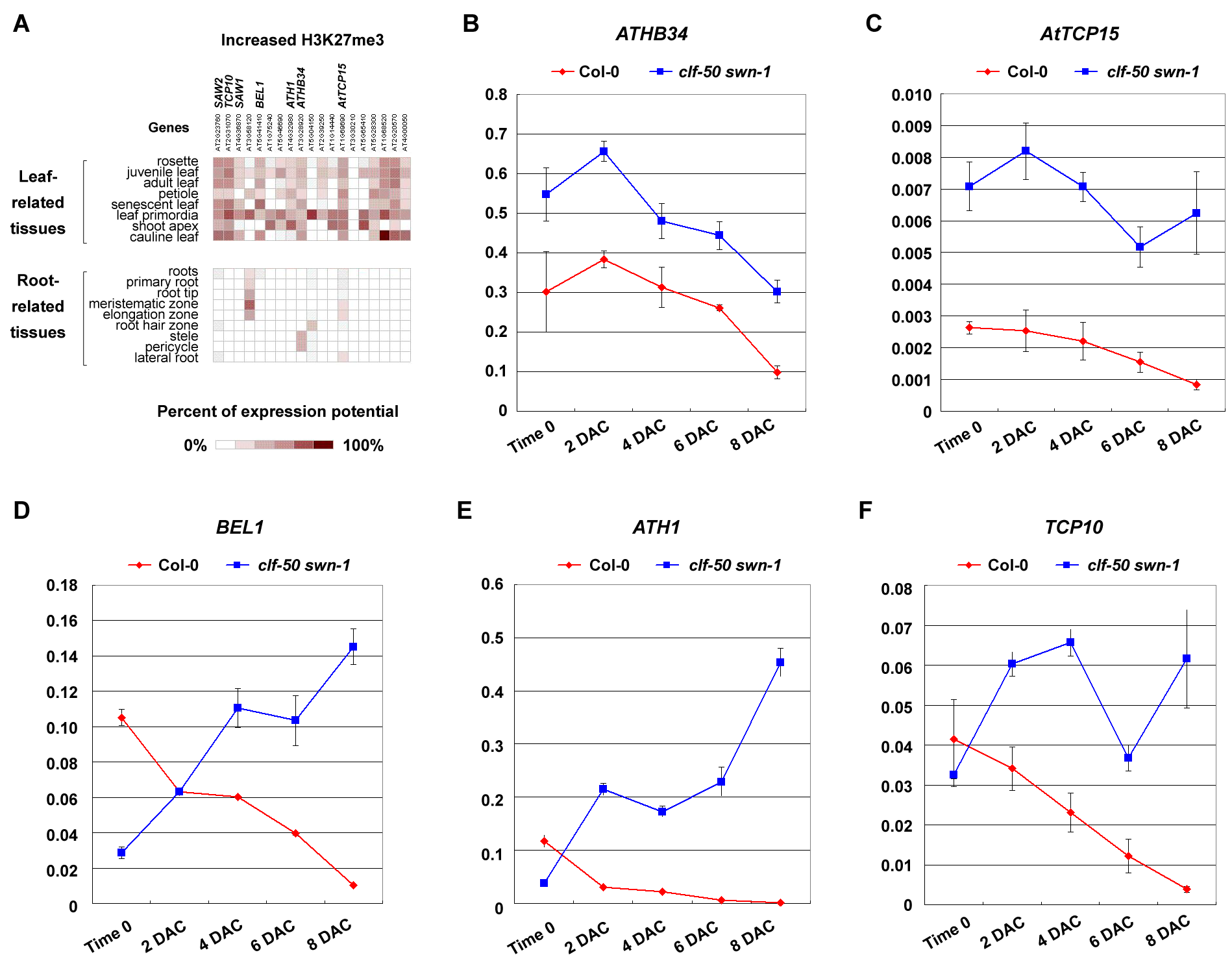

Supplement: Figure S5 — Insufficient repression of the leaf-preferentially expressed regulatory genes in the clf-50 swn-1 double mutant. (A) A total of 19 leaf-preferentially expressed putative transcription factor genes were analyzed, and results from 18 genes were obtained and all showed an insufficient repression pattern in clf-50 swn-1 as compared with that in the wild type. (B–F) Expression patterns of 5 genes, ATHB34 (AT3G28920) (B), AtTCP15 (AT1G69690) (C), BEL1 (AT5G41410) (D), ATH1 (AT4G32980) (E), and TCP10 (AT2G31070) (F), are shown. qRT-PCR was performed using time 0 to 8 DAC leaf explants. Bars show s.e. (TIF) [file pgen.1002911.s005.tif]

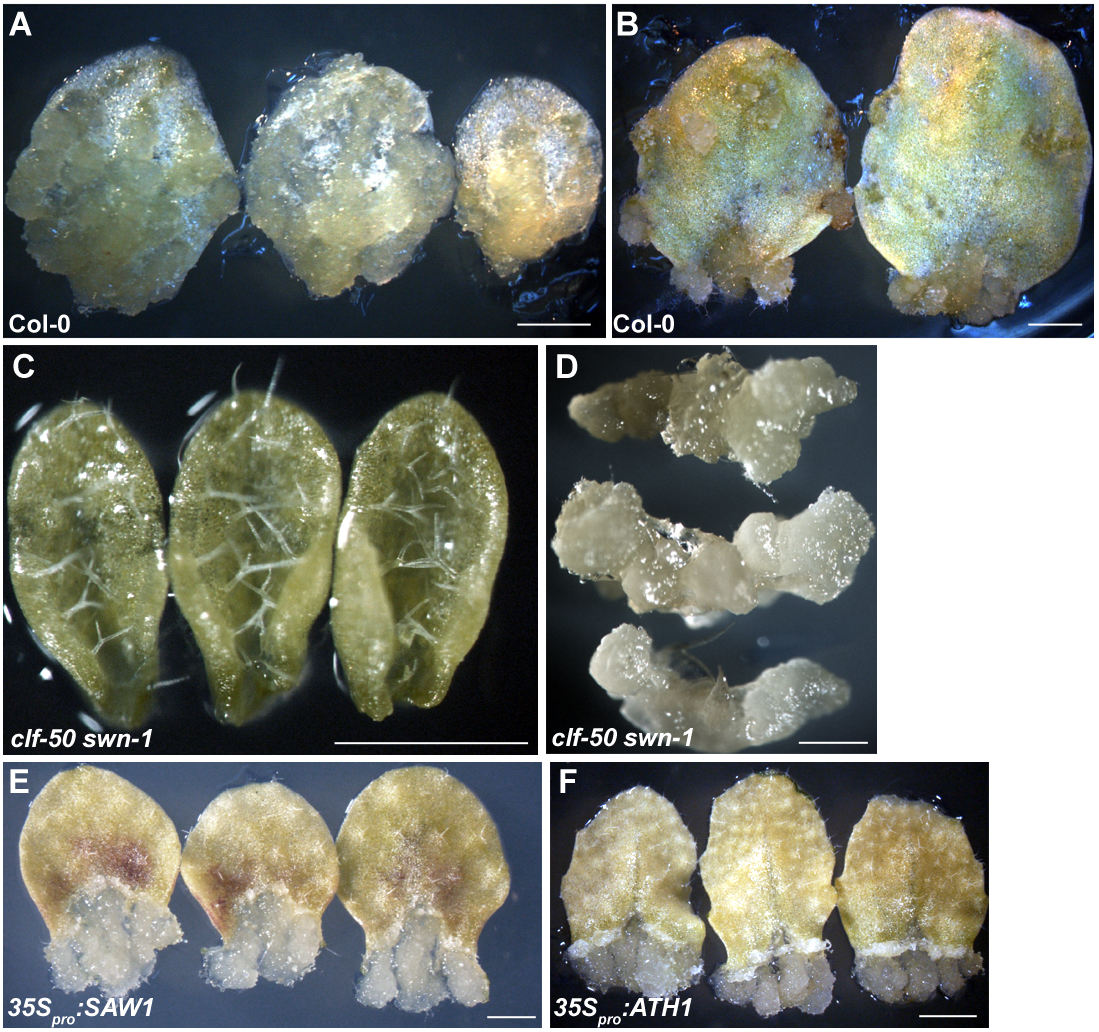

Supplement: Figure S6 — Callus induction using tissues from wild-type, clf-50 swn-1, 35Spro:SAW1, and 35Spro:ATH1 plants. (A and B) Cotyledons (A) and leaf blades (B) from 44-day-old Col-0 seedlings. (C and D) Leaf blades from 9-day-old (C) and roots from 30-day-old (D) clf-50 swn-1 seedlings. (E and F) Leaf blades from 15-day-old 35Spro:SAW1/Col-0 (E) and 35Spro:ATH1/Col-0 (F) seedlings. For each test, 20 leaf blades or cotyledons were cultured, and all explants exhibited consistent phenotypes, except one clf-50 swn-1 leaf blade formed callus. 35Spro:SAW1 and 35Spro:ATH1 were constructed by cloning the cDNAs encoding the full length proteins driven by the 35S promoter. Bars = 1 mm in (A–C, E and F) and 1 mm in (D). (TIF) [file pgen.1002911.s006.tif]

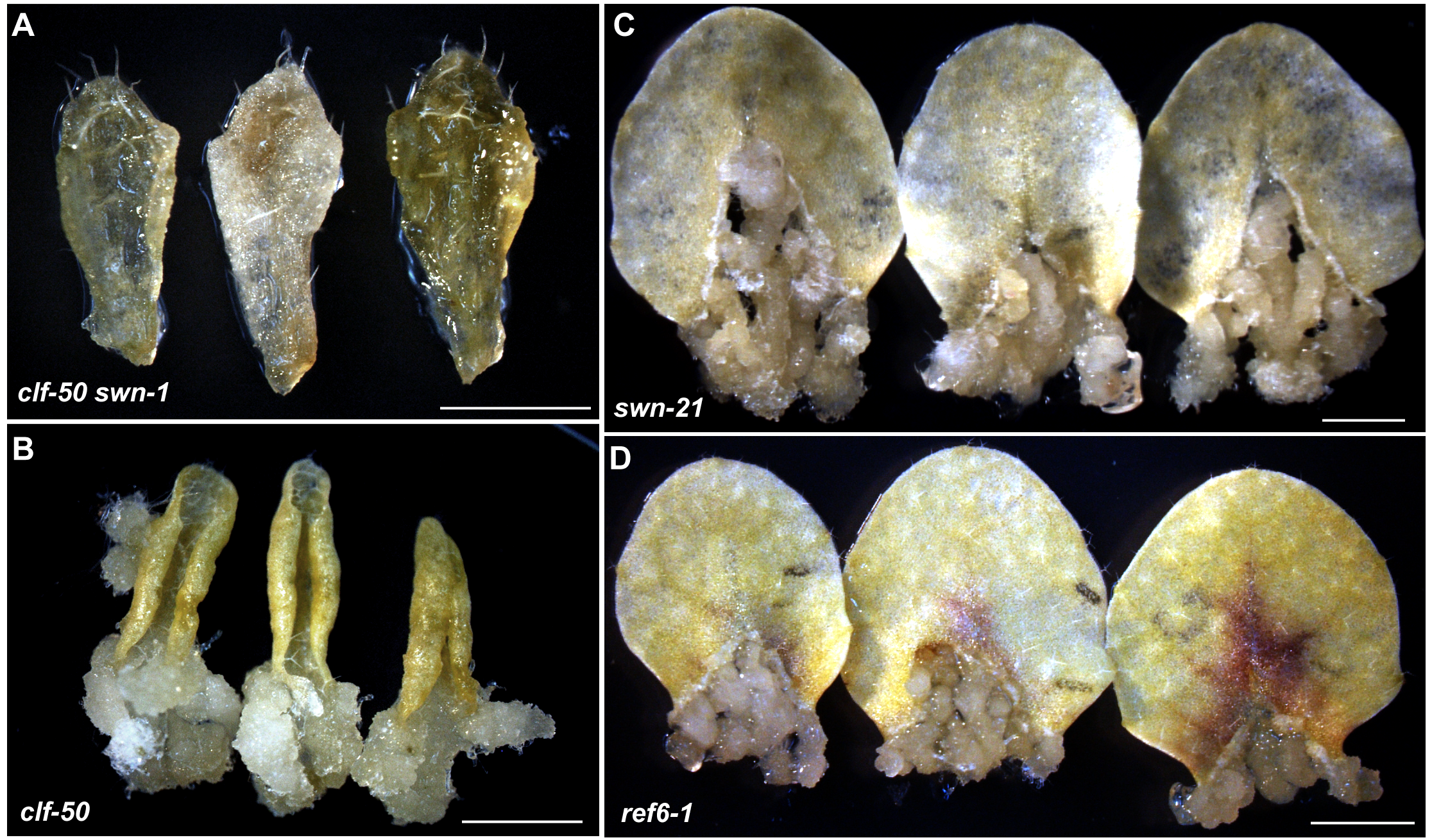

Supplement: Figure S7 — Regeneration abilities of leaf explants of clf-50 swn-1, clf-50, swn-21, and ref6-1. (A) The first two 20-day-old rosette leaves of clf-50 swn-1. (B–D) The third and fourth 20-day-old rosette leaves of clf-50 (B), swn-21 (C) and ref6-1 (D). More than 30 blades were tested for each mutant, and they all exhibited the consistent phenotype. Bars = 1 mm in (A) and 2 mm in (B–D). (TIF) [file pgen.1002911.s007.tif]
