# Supplementary material for: Reprogramming of H3K27me3 Is Critical for Acquisition of Pluripotency from Cultured Arabidopsis Tissues
Source: PLoS Genet. 2012 Aug 23;8(8):e1002911. doi: 10.1371/journal.pgen.1002911 (PMC3426549; doi:10.1371/journal.pgen.1002911)
Supplement: Table S3 — List of primers used in this study. (DOC) [file pgen.1002911.s010.doc]

**Supporting Information**

**Table S3. List of primers used in this study.**

| Experiments | Primers | Sequence (5’🡪3’) |
| --- | --- | --- |
| ChIP | | |
|  | GH3.2-1-F | GTTAATCTCTCTTTCACGGAC |
|  | GH3.2-1-R | CGGATGAGAATCCGGAATTGC |
|  | IAA2-1-F | GTACGAGAAAGTCAACGAGC |
|  | IAA2-1-R | ACGCAAGAAACCTCTTGTTC |
|  | SAW1-1-F | GCTATGCACCAACAATGACC |
|  | SAW1-1-R | CCCATAATACCCTCATCTGTC |
|  | SAW2-1-F | GTGGTGGTTTGGTTCATTGAG |
|  | SAW2-1-R | GAAATGTCGGCTGATATCAAC |
|  | WOX5-6-F | CTGCTGGTGTCATACGTACTG |
|  | WOX5-6-R | GTACAACTCATACATGCCTCAG |
|  | SHR-1-F | GTAACGTAGTCGCGGAATGTC |
|  | SHR-1-R | CGCATCCTAAATCAACAGAC |
|  | AG-4-F | GGCTTTGGAGCAGCAATCAC |
|  | AG-4-R | GCAAACCATTTCTACGTTTGC |
|  | | |
| qRT-PCR | | |
|  | GH3.2-F | CGTATATCCAACGGCGATTG |
|  | GH3.2-R | GTAATAACTCGTGAGAACCGG |
|  | IAA2-F | GTACGAGAAAGTCAACGAGC |
|  | IAA2-R | CTCTAACGCTTTGAGAAGCTC |
|  | SAW1-F | CACCTATTGGCTCGACAGAC |
|  | SAW1-R | CGTTGGAGTTTGTGAAGTGG |
|  | SAW2-F | GCGATGCTGATAAGCACCTC |
|  | SAW2-R | GGAATGAAGAGTCGTTTTCG |
|  | WOX5-F | CGGCAAGATAGAGAGCAAG |
|  | WOX5-R | CGAAGATCTAATGGCGGTGG |
|  | SHR-F | GGGTTTGCTTCGAGTCATGG |
|  | SHR-R | GGCTGATCTCTCCAACAAAG |
|  | ATHB34-F | CGTGACGAAGACGAAGTTCG |
|  | ATHB34-R | GAAGACGACGAGGCGTTTAC |
|  | ATTCP15-F | GTTTACAACCCCAACAGTGG |
|  | ATTCP15-R | CTAGGAATGATGACTGGTGC |
|  | BEL1-F | CTTGTTGACGCTCATCCTTG |
|  | BEL1-R | GGTTCGACACGGATCAACTG |
|  | ATH1-F | CTATGTTCCACCAGCATTGC |
|  | ATH1-R | GGCATTCGAAGAGTTGGTCC |
|  | TCP10-F | GTTTCGTTCTTGGGATCATC |
|  | TCP10-R | CATGAATTTGAACCTCCATGG |
|  | ACTIN-F | TGGCATCA(T/C)ACTTTCTACAA |
|  | ACTIN-R | CCACCACT(G/A/T)AGCACAATGTT |
|  | | |
| Molecular cloning | | |
|  | pSHR-F | AAGCTTAGAAGCAGAGCGTGGGGTTTC |
|  | pSHR-R | GGATCCTTTTTTTTTTTAATGAATAAGAAAATG |
|  | pWOX5-F | GTCGACGCCAACGTTACAACTTACAAC |
|  | pWOX5-R | GGATCCGTTCAGATGTAAAGTCCTCAAC |
